# Model-based Bayesian inference of brain oxygenation using quantitative BOLD

**DOI:** 10.1016/j.neuroimage.2019.116106

**Published:** 2019-08-17

**Authors:** Matthew T. Cherukara, Alan J. Stone, Michael A. Chappell, Nicholas P. Blockley

**Affiliations:** a Institute of Biomedical Engineering, Department of Engineering Science, University of Oxford, Oxford, UK; b Wellcome Centre for Integrative Neuroimaging, FMRIB, Nuffield Department of Clinical Neurosciences, University of Oxford, Oxford, UK

**Keywords:** Quantitative BOLD, Asymmetric spin echo, Bayesian inference, Oxygen metabolism, Oxygen extraction fraction

## Abstract

Streamlined Quantitative BOLD (sqBOLD) is an MR technique that can non-invasively measure physiological parameters including Oxygen Extraction Fraction (OEF) and deoxygenated blood volume (DBV) in the brain. Current sqBOLD methodology rely on fitting a linear model to log-transformed data acquired using an Asymmetric Spin Echo (ASE) pulse sequence. In this paper, a non-linear model implemented in a Bayesian framework was used to fit physiological parameters to ASE data. This model makes use of the full range of available ASE data, and incorporates the signal contribution from venous blood, which was ignored in previous analyses. Simulated data are used to demonstrate the intrinsic difficulty in estimating OEF and DBV simultaneously, and the benefits of the proposed non-linear model are shown. *In vivo* data are used to show that this model improves parameter estimation when compared with literature values. The model and analysis framework can be extended in a number of ways, and can incorporate prior information from external sources, so it has the potential to further improve OEF estimation using sqBOLD.

## 1 Introduction

Quantitative measurements of the BOLD signal can be used to non-invasively construct maps of parameters related to brain metabolism, which have been shown to be useful in clinical assessment of stroke (Seiler et al., 2017), as well as for understanding baseline healthy brain function (Rodgers et al., 2016). Oxygen extraction fraction (OEF) is of particular relevance as a measure of activity, and can be combined with measurements of cerebral blood flow to directly quantify metabolism. The quantitative BOLD (qBOLD) model (Yablonskiy and Haacke, 1994) relates oxygen extraction fraction and deoxygenated blood volume (DBV) to the measured reversible transverse relaxation rate $R_{2}^{\overset{\overset{’}{}}{}}$ (where $R_{2}^{\overset{\overset{’}{}}{}}$ = $R_{2}^{*}$ − *R*_2_). Streamlined quantitative BOLD (sqBOLD - Stone and Blockley, 2017) uses a simplified version of this model with $R_{2}^{\overset{\overset{’}{}}{}}$-weighted measurements made using an asymmetric spin echo pulse sequence (ASE - Wismer et al., 1988) to provide fast and robust quantitative BOLD measurements. Simplification of the qBOLD model is made possible by minimising the signal contribution of confounding factors during data acquisition, such as macroscopic magnetic field gradients using GESEPI (Yang et al., 1998) and cerebrospinal fluid (CSF) using FLAIR (Hajnal et al., 1992).

sqBOLD uses the following stepwise process: first, $R_{2}^{\overset{\overset{’}{}}{}}$ is measured from ASE volumes with a large spin echo displacement *τ*; then, DBV is calculated from the mismatch between a linear exponential model fit and the measured spin echo data; finally, OEF is calculated as a function of the ratio of $R_{2}^{\overset{\overset{’}{}}{}}$ and DBV. This procedure could propagate noise resulting in poor signal-to-noise ratio (SNR), and could introduce bias into the estimates. A curve-fitting approach in which multiple parameters are fitted simultaneously to a more complete qBOLD model (He and Yablonskiy, 2007; Simon et al., 2016) could overcome the limitations imposed by fitting the linear model required in the existing analysis.

Non-linear model fitting, as is required for the qBOLD model, is notoriously challenging on data with limited SNR. Bayesian methods provide a valuable framework for approaching this problem and have been used in similarly challenging applications such as for perfusion estimation from Arterial Spin Labelling (Chappell et al., 2009). In particular, Bayesian methods allow for the incorporation of prior knowledge about physiological parameters (such as might be obtained from both imaging and non-imaging sources), which are used to regularise the fitting. Bayesian methods also give an estimate of the uncertainty in model parameters, which could provide extra information about the validity or interpretability of the results.

In this paper, simulated ASE data are used to evaluate different versions of the qBOLD model in a Bayesian framework with the primary aim of selecting a model that could provide the most reliable estimates of OEF. *In vivo* data are then used to compare a fully model-based parameter estimation procedure against the existing method.

## 2 Theory

### 2.1 Quantitative BOLD model

The qBOLD model, proposed by Yablonskiy and Haacke (1994) describes how transverse magnetisation in bulk tissue changes in the presence of susceptibility altering objects. In this case, those objects are blood vessels whose susceptibility difference is proportional to OEF, and the protons in the surrounding tissue are assumed to be relatively stationary with respect to the vessels (the static dephasing regime). When modelled as randomly oriented, infinitely long cylinders with uniform magnetic susceptibility, the signal originating from the surrounding tissue, as a function of time, is given by (An and Lin, 2003):

(1) $$S^{t}\left(t\right)=\text{exp}\left(-R_{2}^{t}\cdot t\right)\cdot \text{exp}\left(-DBV\cdot \int_{0}^{1}\frac{\left(2+u\right)\sqrt{1-u}}{3\cdot u^{2}}\left(1-J_{0}\left(\frac{3}{2}\delta \omega tu\right)\right)du\right)$$

where $R_{2}^{\overset{t}{\overset{}{}}}$ is the irreversible transverse relaxation rate of bulk tissue, *J*_0_(*x*) is the zero-order Bessel function of *x*, and *δω* is the characteristic frequency, given by:

(2) $$\delta \omega =\frac{4}{3}\pi \cdot \gamma B_{0}\cdot \Delta \chi_{0}\cdot Hct\cdot OEF$$

where *γ* is the proton gyromagnetic ratio, *Δχ*_0_ is the susceptibility difference between the tissue and deoxyhaemoglobin contained within blood vessels, and *Hct* is the fractional haematocrit. This model assumes that deoxyhaemoglobin is the dominant source of magnetic susceptibility. Therefore, the presence of an additional source of susceptibility, such as myelin in white matter or iron deposited in deep grey matter structures, will confound estimates of OEF and DBV. This has previously been shown to result in the overestimation of $R_{2}^{\overset{\overset{’}{}}{}}$ and DBV (Stone and Blockley, 2017). Hence the following analyses are restricted to grey matter.

#### 2.1.1 One compartment model

Under ASE, a refocussing pulse is applied at time (*TE* − *τ*) /2, leading to an effective echo time of *TE* − *τ* (Wismer et al., 1988). The signal is read-out at time *TE* (which is constant across all echoes), and so can be expressed as a function of *τ*. The integral in Equation (1) can be replaced by two asymptotic forms (Yablonskiy and Haacke, 1994):

(3) $$S^{t}\left(\tau \right)=\{\begin{array}{cc} S_{0}\cdot \text{exp}\left(-R_{2}^{t}\cdot TE\right)\cdot \text{exp}\left(-\frac{3}{10}\cdot DBV\cdot {\left(\delta \omega \cdot \tau \right)}^{2}\right) & \left|\tau \right|<t_{c}\\ S_{0}\cdot \text{exp}\left(-R_{2}^{t}\cdot TE\right)\cdot \text{exp}\left(DBV-DBV\cdot \delta \omega \cdot \tau \right) & \left|\tau \right|>t_{c} \end{array}$$

where the characteristic time *t_C_* depends on *δω*, and the factor *S*_0_ controls for constant terms, including R_2_ decay and inter-voxel differences in signal. Given that $R_{2}^{\overset{\overset{’}{}}{}}=DBV\cdot \delta \omega ,$ this equation can also be written in terms of $R_{2}^{\overset{\overset{’}{}}{}}$, removing explicit dependence on OEF:

(4) $$S^{t}\left(\tau \right)=\{\begin{array}{cc} S_{0}\cdot \text{exp}\left(-R_{2}^{t}\cdot TE\right)\cdot \text{exp}\left(-\frac{3}{10}\cdot \frac{{\left(R_{2}^{\overset{\overset{’}{}}{}}\;\cdot \;\tau \right)}^{2}}{\text{DBV}}\right) & \left|\tau \right|<t_{c}\\ S_{0}\cdot \text{exp}\left(-R_{2}^{t}\cdot TE\right)\cdot \text{exp}\left(\text{DBV}-R_{2}^{\overset{\overset{’}{}}{}}\;\cdot \;\tau \right) & \left|\tau \right|>t_{c} \end{array}.$$

These equations are two forms of a normalized one-compartment (1C) qBOLD model. The latter can be used to calculate OEF by rearranging Equation (2):

(5) $$OEF=\frac{3\cdot R_{2}^{\overset{,}{}}}{4\pi \cdot \gamma B_{0}\cdot \Delta \chi_{0}\cdot Hct\cdot DBV}$$

#### 2.1.2 Log-linear model

The sqBOLD technique uses the long-*τ*, $R_{2}^{\overset{\overset{’}{}}{}}$-dependent model (Equation (4b)) to fit $R_{2}^{\overset{\overset{’}{}}{}}$ and DBV (Stone and Blockley, 2017). $R_{2}^{\overset{\overset{’}{}}{}}$-weighted images with *τ* > *t_c_* can be fit to a log-transformed model (L-model):

(6) $$\text{In}S^{t}\left(\tau \right)=-R_{2}^{\overset{\overset{’}{}}{}}\cdot \tau +\text{In}\;S_{SE}^{t}+\text{DB}V.$$

Here $R_{2}^{\overset{\overset{’}{}}{}}$ can be calculated as the gradient of ln*S^t^*(*τ*), and DBV can be obtained by subtraction of ln $S_{\text{SE}}^{\overset{t}{\overset{}{}}}$ from the intercept, where $S_{\text{SE}}^{\overset{t}{\overset{}{}}}$ is the signal measured at the spin echo (*τ* = 0). OEF can then be calculated using Equation (5).

#### 2.1.3 Two compartment model

The qBOLD model can be extended to include the signal which originates from venous blood. Several models have been proposed which describe this intravascular signal. The first assumes that the blood signal *S^b^* decays monoexponentially, and is reversible with respect to the spin echo (Simon et al., 2016):

(7) $$S^{b}\left(\tau \right)=\text{exp}\left(-R_{2}^{b}\cdot TE\right)\cdot \text{exp}\left(-R_{2}^{b*}\cdot \left|\tau \right|\right)$$

where $R_{2}^{\overset{\text{b}}{\overset{}{}}}$ and $R_{2}^{b*}$ are functions of OEF (and *Hct*). This does not properly consider the dephasing effects inside the blood vessel, which consists of a bulk (plasma) containing susceptibility altering objects (red blood cells). The intravascular dephasing can be parametrised in terms of Fresnel functions (Sukstanskii and Yablonskiy, 2001; Yablonskiy et al., 2012):

(8) $$S^{b}\left(\tau \right)=\text{exp}\left(-R_{2}^{b}\cdot TE+i\frac{\delta \omega \cdot \tau }{2}\right)\cdot \frac{C\left(\eta \right)-iS\left(\eta \right)}{\eta }$$

where *C*(*η*) and *S*(*η*) are Fresnel functions, and

(9) $$\eta ={\left(\frac{3\cdot \delta \omega \cdot \left|\tau \right|}{\pi }\right)}^{1/2}.$$

A recently proposed analytical model describes the blood signal under the motional narrowing regime. This is valid because the size of a red blood cell is significantly smaller than the distance a spin in the plasma will diffuse during time TE. The signal in this regime is (Berman et al., 2018):

(10) $$S^{b}\left(\tau \right)=\text{exp}\left(-R_{2}^{b,true}\cdot TE\right)\cdot \text{exp}\{-\frac{\gamma^{2}}{2}G_{0}t_{D}^{2}\cdot (\frac{TE}{t_{D}}+\sqrt{\frac{1}{4}+\frac{TE}{t_{D}}}+\frac{3}{2}-2\sqrt{\frac{1}{4}+\frac{TE+\tau }{t_{D}}}-2\sqrt{\frac{1}{4}+\frac{TE-\tau }{t_{D}}})\}$$

Here characteristic diffusion time *t_D_* = *R_rbc_*/*D_b_* where *R_rbc_* = 2.6 μm is the characteristic size of red blood cells and *D_b_* = 2 μm^2^ms^−1^ is their rate of diffusion, $R_{2}^{b\mathrm{true}}$ is the intrinsic *R*_2_ of fully oxygenated blood and *G*_0_ is the mean square field inhomogeneity in blood (Berman et al., 2018):

(11) $$G_{0}=\frac{4}{45}\cdot Hct\cdot \left(1-Hct\right)\cdot {\left(4\pi \cdot B_{0}\cdot OEF\cdot \Delta \chi_{0}\right)}^{2}.$$

The total signal measured from a voxel in this two-compartment model (2C) is the sum of the signal from each compartment, weighted by their apparent volume fractions. Apparent DBV, denoted *ζ*’, is given by:

(12) $$\zeta^{\overset{\overset{’}{}}{}}=m_{b}\cdot n_{b}\cdot DBV$$

where *m_b_* is the steady-state magnetisation of the blood (which depends on its *T*_1_ and the sequence parameters TR, TE, and TI), and *n_b_* is the relative spin density of blood (He and Yablonskiy, 2007). Total 2C signal is therefore given by:

(13) $$S^{TOTAL}\left(\tau \right)=S_{0}\cdot \left(\zeta^{\overset{\overset{’}{}}{}}\cdot S^{b}\left(\tau \right)+\left(1-\zeta^{\overset{,}{}}\right)\cdot S^{t}\left(\tau \right)\right)$$

where *S*_0_ is the signal without any transverse relaxation (at *t* = 0). This can be used as a forward model in a Bayesian framework to infer parameter distributions for either OEF and DBV, or $R_{2}^{\overset{\overset{’}{}}{}}$ and DBV (with OEF calculated from those parameters afterward). A model such as this expected to have most value in voxels which have a relatively small blood volume and where this additional compartment can correct for the presence of intravascular signal. In the presence of larger blood volume fractions, whilst the intravascular signal would be appropriate, the assumption of the extravascular signal model (Eq. (1)) would be invalidated (Yablonskiy and Haacke, 1994).

### 2.2 Bayesian inference

Bayesian inference is built upon Bayes’ theorem, which defines how, for a model ℳ with parameters *Θ*, a posterior probability distribution of those parameters can be formed by combining prior beliefs about the parameter with some observed data *Y*:

(14) $$P\left(\Theta |Y,ℳ\right)=\frac{P\left(\Theta |ℳ\right)P\left(Y|\Theta ,ℳ\right)}{P\left(Y|ℳ\right)}$$

where *P*(*Θ*|*Y*, ℳ) is the posterior probability of the parameters, *P*(*Θ*|ℳ) is the prior, *P*(*Θ*|*Y*, ℳ) is the likelihood of the data, given the parameters and the model, and *P*(*Θ*|ℳ) is the evidence for the data, given the model. Often, analytically evaluating the posterior is impossible. It can, however, be easily sampled in a uniform pattern using a grid search. Though exact, this method is wasteful and prohibitively time-consuming in high dimensional space.

An alternative to sampling the true posterior is to approximate it, then analytically solve the approximation. A variational Bayesian (VB) inference scheme has been developed for non-linear signal models similar to those considered here and applied to imaging data to analytically calculate approximate posterior parameter distributions (Attias, 2000; Chappell et al., 2009).

This is significantly faster than sampling based methods, and still allows prior information to be used to generate more physically reasonable estimates from noisy data (Chappell et al., 2009). The priors take the form of Gaussian distributions, with specified means *μ*_0_ and standard deviations *σ*_0_. These values being chosen based on prior knowledge of typical realistic parameter values (including the possibility of pathological variation), but not so as to bias the results away from what the underlying data would predict. Priors can also be used to enforce physically reasonable conditions such as spatial smoothness by means of a spatial prior distribution (Groves et al., 2009).

## 3 Material and methods

### 3.1 Simulations

Simulated qBOLD data were used to examine whether a Bayesian model-based approach was able to estimate OEF more accurately than least-squares fitting to a simplified log-linear model. In MATLAB (MathWorks, Natick, MA), simulated two-compartment ASE qBOLD signals were generated using Equations (1), (10) and (13). Signals were simulated with 50 OEF values from 20% to 70% (which includes the expected range of healthy values (Marchal et al., 1992)) and 50 DBV values from 0.3% to 15% (based on the expected range of total blood volume (Roland et al., 1987)), for a total of 2500 artificial ASE signals. Gaussian noise was added to simulate 7 SNR values between 5 and 500. Each signal consisted of 24 $R_{2}^{\overset{\overset{’}{}}{}}$-weighted images with spin-echo offsets *τ* in steps of 4 ms between −28 ms and +64 ms. This optimised range of *τ* values were chosen to maximise the SNR of the $R_{2}^{\overset{\overset{’}{}}{}}$ parameter estimate (Stone and Blockley, 2017). Fractional haematocrit *Hct* was set at 0.40, the literature value for general circulation (Nicoll et al., 2012), which is similar to what has been used in other related studies (Simon et al., 2016; Berman and Pike, 2017; Lee et al., 2018). Previous studies have used lower values such as 0.34 (He and Yablonskiy, 2007; Stone and Blockley, 2017), in accordance with the Fahraeus effect (Fahraeus and Lindqvist, 1931), which suggests that *Hct* in small vessels (including capillaries and venules) is lower than in general circulation. This effect has been quantified as a reduction of between 14% (Calamante et al., 2016) and 25% (Sakai et al., 1985). It is not clear to what extent the range of *Hct*s present throughout the brain affect the qBOLD signal, or whether an average value can be assumed throughout. Other physiological and sequence parameters were held to constant values, which are given in Table 1.

In order to assess the differences between the OEF-based and $R_{2}^{\overset{\overset{’}{}}{}}$-based models (Equations (3) and (4) respectively), a single simulated dataset (OEF =40%, DBV = 3%) was analysed in a grid search scheme. The posterior probability of each pair of values in the intervals *OEF* ∈ {20%; 70%} and *DBV* ∈ {0:3%; 15%}, given the 2C model, was evaluated. The OEF-based and $R_{2}^{\overset{\overset{’}{}}{}}$-based models were compared in their accuracy of DBV estimation by marginalizing over OEF and $R_{2}^{\overset{\overset{’}{}}{}}$ respectively. Whilst the grid search technique is prohibitive for analysis of *in vivo* data, it enables direct comparison of parameter distributions to determine separability. Grid searches were used to determine which of the OEF-based or $R_{2}^{\overset{\overset{’}{}}{}}$-based models were more suitable, based on whether OEF-DBV or $R_{2}^{\overset{\overset{’}{}}{}}$-DBV distributions were more separable.

The full set of simulated data were analysed in a VB scheme, using the Fast ASL and BOLD Bayesian Estimation Routine (FABBER) (Chappell et al., 2009; Groves et al., 2009, Woolrich and Behrens, 2006). The 1C and 2C models were used, with parameters DBV and either OEF or $R_{2}^{\overset{\overset{’}{}}{}}$. Prior means *μ*_0_ were taken from the literature for healthy subjects (Derdeyn et al., 2001; Roland et al., 1987) and are presented in Table 2. The effect of different *μ*_0_ and *σ*_0_ were investigated in order to choose values that would not bias the results in the case of very different (e.g. pathological) true values.

VB inference was performed using the 2C model with a range of *σ*_0_ (between 10^−1/2^ and 1000) for each parameter at the highest simulated SNR (SNR = 500), with *μ*_0_ fixed to the values in Table 2. The absolute difference between the estimate of each parameter and its true value was then computed and averaged over all simulated pairs of OEF and DBV values. In order to minimise the risk of biasing the parameter estimates, the least precise prior which still resulted in a low error was chosen for each parameter, and used in all later inference. Once appropriate precisions were chosen, extreme values of *μ*_0_ were tested to ensure that the chosen prior mean did not significantly affect the results. $\mu_{0}\left(R_{2}^{,}\right)=\left\{3s^{-1},7s^{-1},15s^{-1}\right\}$ and *μ*_0_(*DBV*) = {0:5%; 3:6%; 10%} were tested, and errors in estimates of DBV and $R_{2}^{\overset{\overset{’}{}}{}}$ calculated as above.

Least-squares regression (LS), and VB inference, using each model (L, 1C, and 2C), was performed on the same data, for all OEF-DBV pairs and SNR values. The absolute error between true and estimated OEF and DBV values were averaged across all OEF and DBV values for each SNR, in order to assess the utility of a more complete model, and the effect of noise on estimates. These were compared at the voxel level using two-way ANOVA and Tukey-Kramer (honestly significant difference) pairwise comparisons.

Another aspect of the qBOLD model was tested using simulated data. The point at which the asymptotic models transition, *t_C_* (Equations (3) and (4)) was defined originally as *t_C_* = 1.5/*δω* (Yablonskiy and Haacke, 1994), whereas, more recently, Lee et al. (2018) used *t_C_* = 1/ *δω*. Since the model itself is phenomenological, parameters such as *t_C_* should be chosen so that the asymptotic model most closely matches the analytical version. For all OEF-DBV pairs, in the absence of noise, the analytical qBOLD model (Equation (1)) was compared with the asymptotic model (Equation (3)), and an optimal value for the transition constant *τ* · *δω* was found using a golden section search (MATLAB’s fminbnd).

In light of recent efforts to reduce the scan duration for application in clinical research (Stone et al., 2019), a supplementary analysis was performed using alternative acquisition parameters. The acquisition consisted of 11 $R_{2}^{\overset{\overset{’}{}}{}}$-weighted images with spin-echo offsets *τ* in steps of 8 ms between −16 ms and +64 ms, which represents a scan time reduction of 54%. This data was formed by retroactively removing *τ* values from already-simulated data.

### 3.2 Data collection

All imaging was performed on a 3 T S Verio system (Siemens Healthineers, Erlangen, Germany) using the body transmit coil and the vendors 32-channel receive coil. Subjects were scanned under a technical development protocol agreed with local ethics and institutional committees.

Scan data from seven healthy participants (aged 24–32, 4 female), which was collected as part of a previous study (Stone and Blockley, 2017), were used to compare non-linear model inference using VB against a linear-least-squares fitting procedure *in vivo*.

For each subject, a GESEPI ASE (GASE) scan (Blockley and Stone, 2016) with FLAIR preparation (TI_FLAIR_ = 1210 ms) was acquired, with TR/TE = 3000/74 ms, 64 × 64 matrix, ten slabs, 3.75 × 3.75 × 5 mm resolution, and 24 *τ* values ranging from −28 ms to 64 ms in steps of 4 ms. Each 5 mm slab was constructed by averaging four 1.25 mm slices (acquired as 8 k-space partitions including 100% partition oversampling to reduce aliasing) to correct for macroscopic field gradients. Total acquisition time was 9 min 36 s. Data were acquired as part of a previous study (Stone and Blockley, 2016 - see Appendix: Data Access Statement). The GESEPI acquisition compensates for the effect of macroscopic magnetic field gradients by effectively applying multiple z-shim levels and integrating them using a Fourier transform, which is equivalent to the acquisition of several thin partitions in a 3D acquisition (Yang et al., 1998).

### 3.3 Image analysis

Data pre-processing was performed in FSL (Jenkinson et al., 2012). GASE data were motion corrected using MCFLIRT (Jenkinson et al., 2002), and smoothed with a Gaussian kernel (*σ*=0.85 mm). This sub-voxel smoothing was chosen to reduce the impact of noisy voxels, while maintaining the effective spatial resolution of the parameter estimates. The spin-echo volumes (*τ* = 0) were segmented using FAST (Zhang et al., 2001) to create grey matter masks that were used to define the region of interest. Grey matter masks were obtained by thresholding the segmented ASE data at 60%, to exclude voxels where partial volumes of white matter or nulled CSF, where the assumptions of the qBOLD model are violated, may severely affect the signal. This approach was chosen over segmenting the MPRAGE images because it produced more consistent results, and does not require registration from MPRAGE-space to GASE-space.

Grey matter SNR was calculated for each subject across all *τ* values, in order to determine the approximate range of values from simulation that would be most appropriate. This was done by dividing the mean voxel intensity values within the grey matter mask by the standard deviation of values outside the head.

*In vivo* ASE data were analysed using the L model, implemented in MATLAB, and the $R_{2}^{\overset{\overset{’}{}}{}}$-DBV 1C and 2C models in VB both with and without spatial regularization. The 1C and 2C models were fit to data for all 24 *τ* values; whereas the L model only applies to *τ* = 0 and *τ* > *t_C_* (for a total of 14 data points). In addition to the motional narrowing model for intravascular signal (Equation (10)), a supplementary analysis of the linear and powder models (Equations (7) and (8)) was also performed. Inference was performed using a 2C model incorporating each different intravascular signal for all subjects (without spatial regularization) in order to compare their impact on final parameter estimates.

Priors and initial posteriors were defined using literature mean values and the standard deviations determined from testing simulated data (see Section 4.1). These are given in Table 2. Mean grey matter parameter estimates were compared for each analysis method, for each subject and across the group. Under spatial regularization, each voxel’s prior was defined by aggregating the posterior distributions from the 6 adjacent voxels, across 10 iterations. This imposes a degree of spatial smoothness in the parameter distributions.

To determine similarity, estimates were subjected to a two-way ANOVA test to determine whether the group means for each method were equal. In the case of significantly different group means, the Tukey-Kramer (honestly significant difference) method was used to perform pairwise comparisons.

In a Bayesian framework, it is also possible to assess goodness of fit using the model evidence term (*P*(*Y*|ℳ) in Equation (14)). The VB routine aims to minimise the Kullback Liebler divergence between the (intractable) true posterior and a (tractable) approximate posterior. Since *P*(*Y*|ℳ) is constant for a given model, it can be shown (by rearranging Equation (14)) that this minimization is equivalent to maximizing the free energy of the approximate posterior (Attias, 2000; Chappell et al., 2009). A model that results in a higher average free energy across grey matter therefore provides a better explanation of the data. The median grey matter free energy values for each method were analysed across subjects with the same two-way ANOVA test, and Tukey-Kramer pairwise comparisons. This was to ensure that applying additional constraints, such as spatial smoothness in parameters, did not negatively impact the fitting.

A supplementary comparison of L, 1C, and 2C models (using VB inference) was also carried out on the same data, analysing only 11 of the originally acquired 24 *τ* values, from −16 ms to +64 ms in steps of 8 ms. The resulting grey matter mean parameter values were compared between models, and between the 24-*τ* and 11-*τ* datasets.

To determine the impact of the assumed value of *Hct* = 0:40 (Nicoll et al., 2012), a further supplementary analysis was performed in which the same data (with 24 *τ* values) were analysed with all models with *Hct* = 0:34 (as in He and Yablonskiy, 2007, and Stone and Blockley, 2017). It is expected that in the L and 1C models, *Hct* will act purely as a scaling constant on OEF (Equation (5)); but since it occurs in the intravascular compartment (Equation (11)) its effect on $R_{2}^{\overset{\overset{’}{}}{}}$ and DBV estimates from the 2C model should also be investigated.

## 4 Results

### 4.1 Simulated data

**Fig. 1:**
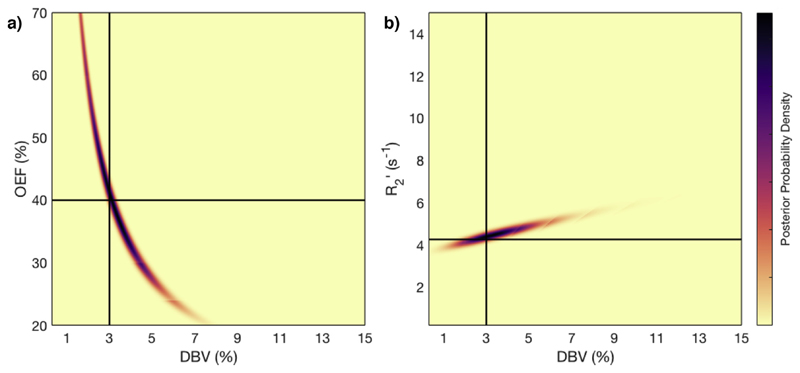
Results of grid-search posterior sampling on simulated ASE qBOLD data, with SNR 50. a) Posterior probability of OEF-DBV parameter pairs, with true (simulated) values shown as solid black lines. b) Posterior probability of $R_{2}^{\overset{\overset{’}{}}{}}$-DBV pairs, using the same data. In the OEF-DBV model, there is a large area of collinearity, and the posterior density distribution does not have a Gaussian-like form. By contrast, the $R_{2}^{\overset{\overset{’}{}}{}}$-DBV model has more separable parameters, and a distribution shape that can more easily be approximated by a multivariate normal distribution, which is a requirement for VB inference as implemented here.

Asymptotic qBOLD models given in terms of OEF and $R_{2}^{\overset{\overset{’}{}}{}}$ were compared using grid search posterior sampling of simulated data. The results of this are shown in Fig. 1. In OEF-DBV space, there is a large region of collinearity between the two parameters, which is likely to prevent accurate estimation of both parameters. In contrast, the $R_{2}^{\overset{\overset{’}{}}{}}$-DBV space has a posterior distribution in which the two parameters are largely separable and also which can more readily be approximated as a multivariate normal distribution within the VB inference method. Marginalizing over these results with respect to OEF and $R_{2}^{\overset{\overset{’}{}}{}}$ yielded DBV likelihood distributions that are directly comparable. The standard deviation in DBV was 1.05% for the OEF-based model, and 1.14% for the $R_{2}^{\overset{\overset{’}{}}{}}$ model, suggesting that there is very little difference between the two in their estimation of DBV. The same analysis of OEF estimates derived from each method yielded a standard deviation of 14.5% for the OEF-based model, and 15.8% for the $R_{2}^{\overset{\overset{’}{}}{}}$ model.

**Fig. 2:**
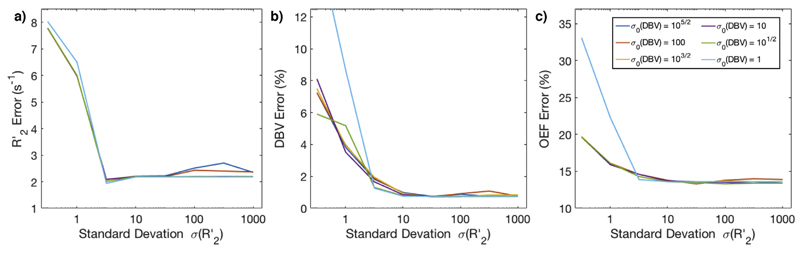
Optimization of prior standard deviations *σ*_0_ based on error in parameter estimates on simulated data. a) The effect of priors on $R_{2}^{\overset{\overset{’}{}}{}}$ error, which is not strongly affected by *σ*_0_, except at $\sigma_{0}\left(R_{2}^{\overset{\overset{’}{}}{}}\right)$ ≤ 1. b) The effect of priors on DBV error, which diverges quickly at low $\sigma_{0}\left(R_{2}^{\overset{\overset{’}{}}{}}\right)$, but is consistently at $\sigma_{0}\left(R_{2}^{\overset{\overset{’}{}}{}}\right)$ ≥ 10. c) The effect of priors on OEF error, which was lowest for $\sigma_{0}\left(R_{2}^{\overset{\overset{’}{}}{}}\right)$ > 1.

Simulated ASE signals were used to compare the L model with the more complicated 1C and 2C models analysed in VB. First, the optimal parameters for VB inference were determined by testing a range of standard deviations for the priors on $R_{2}^{\overset{\overset{’}{}}{}}$ (between 10^5/2^ and 1000) and DBV (between 10^−5^ and 1). The results of these are shown in Fig. 2. They suggest that $\sigma_{0}(R_{2}^{\overset{\overset{’}{}}{}})=10^{3/2}$ and *σ*_0_(*DBV*) = 10^3/2^ are the least precise values that consistently produce reasonable results, although using an even broader $\sigma_{0}\left(R_{2}^{\overset{\overset{’}{}}{}}\right)$ is not detrimental. Using these *σ*_0_, the choice of prior mean *μ*_0_ was also investigated by testing extreme values. The error in $R_{2}^{\overset{\overset{’}{}}{}}$ and DBV estimates was calculated using each combination of $\mu_{0}\left(R_{2}^{\overset{\overset{’}{}}{}}\right)$ and *μ*_0_(*DBV*), and the standard deviation of these was 0.76 s^−1^ for $R_{2}^{\overset{\overset{’}{}}{}}$, and 0.47% for DBV, showing that the choice of *μ*_0_ does not significantly bias the results.

Simulated data were also used to determine the optimal transition point between the two asymptotic regimes, as a function of *δω*. This was calculated for each simulated OEF and DBV value, in intervals *OEF* ∈ {20%, 70%} and *DBV* ∈ {0:3%, 15%}. Averaging across the entire parameter space, the optimal transition point was found to be 1:76 /*δω*. This value was used in all further analysis of simulated and *in vivo* data, for all models.

**Fig. 3:**
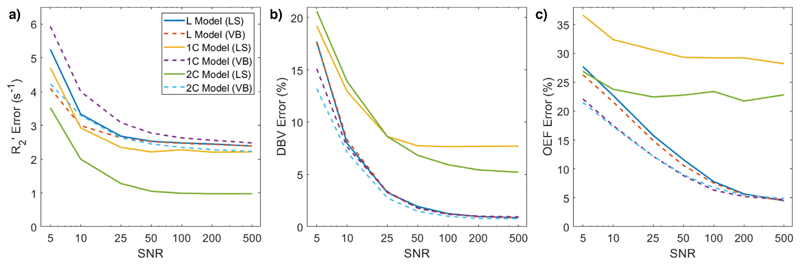
Error in parameter estimates of a) $R_{2}^{\overset{\overset{’}{}}{}}$, b) DBV, and c) OEF as a function of SNR, for each model. Across all SNR levels, the least squares (LS) implementations of the 1C and 2C models performed poorly in estimating DBV and OEF. At SNRs below 100, the variational Bayesian (VB) implementations of the 1C and 2C models estimated OEF significantly (*p* < 0:001) more accurately than other models.

Using optimised parameters, the simulated ASE signals were analysed using each of the three models (L, 1C, and 2C) at 7 SNR values. Each model was evaluated with both least squares regression (LS) and VB. For each signal, the absolute error between the true value of each parameter and the estimate was calculated. The average errors across the whole parameter space, at each SNR and for each model, are shown in Fig. 3. The 2C model in LS performed better than any other at estimating $R_{2}^{\overset{\overset{’}{}}{}}$. Two-way ANOVA with pairwise comparisons showed that there were not significant differences in estimates of $R_{2}^{\overset{\overset{’}{}}{}}$ between all the other methods. The 1C and 2C models in LS performed significantly worse in estimating DBV, and there were no significant differences between the others. At all tested SNRs except 100, the 1C and 2C models in VB performed significantly (*p* < 0:001) better than the L model, or any LS implementations, at estimating OEF. Apart from at very low SNR (SNR ≤ 10) and at SNR = 500 there were not significant differences in parameter estimates between the LS and VB implementations of the L model.

Across the range of data simulated *OEF* ∈ {20%, 70%} and *DBV* ∈ {0:3%, 15%}, the 1C and 2C models performed similarly well; however, in the high DBV regime (*DBV* > 10%), the 2C model estimated both $R_{2}^{\overset{\overset{’}{}}{}}$ and DBV more accurately than L or 1C models. $R_{2}^{\overset{\overset{’}{}}{}}$ error was 14 ± 3% lower with 2C estimation (*p* < 0:001, based on a one-sample *t*-test), and DBV error was 24 ± 9% lower (*p* < 0:05).

The results of the analysis using alternative acquisition protocol are presented in Supplementary Information. Fig. S1 parallels Fig. 3 displaying the error in estimations of $R_{2}^{\overset{\overset{’}{}}{}}$, DBV, and OEF, as a function of SNR. The error in OEF estimation across all SNRs are higher in the case of fewer *τ* values, but in this case the 2C model offers an even greater improvement over the L model than 1C, especially at lower SNR (SNR ≤ 50).

### 4.2 *In vivo* data

The analysis methods (L model in least squares fitting, and 1C and 2C models in VB with and without spatial regularization) were tested on GASE data from 7 healthy subjects. Computation time for the L model analysis averaged 2.5 s per subject, for the entire volume, and for both 1C and 2C models in VB was 10.3 s each per subject. VB with spatial regularization took, on average, 11.8 s per subject (for 1C and 2C models).

Grey matter SNR was calculated by dividing the mean GASE voxel intensity within grey matter by the standard deviation of the noise in empty space outside the head. Across all subjects and *τ* values, SNR was 89 ± 16.

**Fig. 4:**
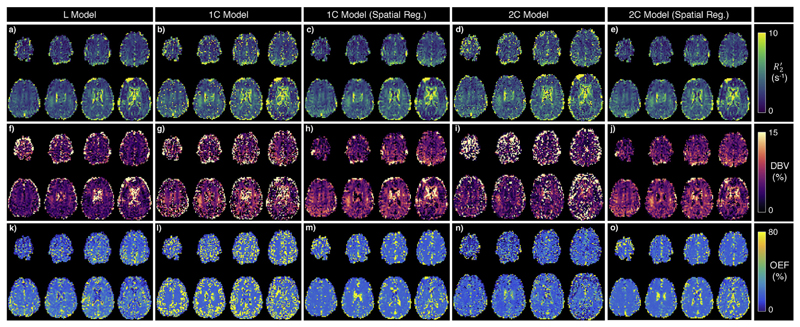
Example parameter maps for a single subject, showing estimated $R_{2}^{\overset{\overset{’}{}}{}}$ (top), DBV (middle) and OEF (bottom), using the L model (first column), 1C model (second column), 1C model with spatial regularization (third column), 2C model (fourth column), and 2C model with spatial regularization (fourth column). For all parameters, the use of spatial regularization drastically reduces the number of bright voxels, where parameters were previously estimated as extreme values.

**Fig. 5:**
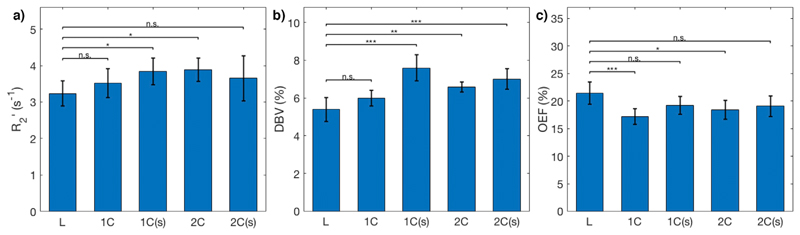
Group (N = 7) average grey matter estimates of a) $R_{2}^{\overset{\overset{’}{}}{}}$, b) DBV, and c) OEF, with error bars indicating inter-subject standard deviation, for the L model, and 1C and 2C models; (s) indicates spatial regularization. Two-way ANOVA and pair-wise comparisons show that the 1C and 2C models produce similar $R_{2}^{\overset{\overset{’}{}}{}}$ estimates as the L model, although the DBV estimates are different except in the non-regularized 1C model. The 1C and 2C models with spatial regularization produce estimates of OEF that are not statistically significantly different from those of the L model.

Grey matter mean parameter values for $R_{2}^{\overset{\overset{’}{}}{}}$, DBV, and OEF for each subject are presented in Table 3, as well as inter-voxel standard deviations of parameter estimates, which account for physiological variation as well as uncertainty in model fitting. Maps from an example subject are shown in Fig. 4, and a group-level comparison is shown in Fig. 5. The group-average grey matter OEF estimate from the L model was 21 ± 2%, the 1C model estimated 17 ± 2% without spatial regularization, and 19 ± 2% with spatial regularization. The 2C model estimated OEF at 18 ± 2% and 20 ± 2% (without and with spatial regularization, respectively). Group-average grey matter DBV was 5.38 ± 0.62%, 5.99 ± 0.43%, and 6.58 ± 0.26% for L, 1C, and 2C models respectively.

At the inter-subject level, the variance in $R_{2}^{\overset{\overset{’}{}}{}}$ improved significantly (based on an F-test with *α* = 0:05) with the 1C model (both with and without spatial regularization) compared to the L model. Group-average grey matter standard deviation of $R_{2}^{\overset{\overset{’}{}}{}}$ was 2.7s^−1^ in least-squares fitting, compared to 1.21s^−1^ under VB. Variances in OEF and DBV were lower in spatially-regularized VB, but were not statistically significant. At the intra-subject level, the improvements of VB were statistically significant in all subjects for $R_{2}^{\overset{\overset{’}{}}{}}$, and in 4 of 7 subjects for DBV and OEF (based on an F-test with 100 degrees of freedom). Spatially regularized VB made a significant improvement in the variances of all parameters across all subjects. This effect can be seen in the relative number of voxels which contain values that are not physically plausible, such as those with OEF or DBVs greater than 100%. Across the group, the proportion of grey matter voxels with OEF estimates greater than 100% was 33.7% under VB without spatial regularization, compared to 8.6% with spatial regularization (under the L model, the proportion was 18.8%). Fig. 4 illustrates this improvement. Using the 2C model also resulted in a decrease in the proportion of voxels with OEFs or DBVs above 100% (17.7% and 8.1% without and with spatial regularization, respectively).

Two-way ANOVA showed statistically significant differences in average grey matter estimates of $R_{2}^{\overset{\overset{’}{}}{}}$ between L model and the 1C and 2C models inferred under VB. There was a significant difference between L model and 1C model estimates of DBV, but not between L and 2C. There were significant differences between the L model and all others (except non-spatially regularized 1C model) in estimates of OEF. For both 1C and 2C, across all parameters, there were no group-level statistically significant differences between estimates made with and without spatial regularization.

Additional analysis of the 2C model was performed using different models of the intravascular signal, which is shown in the Supplementary Material. Fig. S2 parallels Fig. 5, and shows that there is no significant difference in $R_{2}^{\overset{\overset{’}{}}{}}$ or OEF estimation between the three intravascular signal models. There is significant difference in DBV estimation, and the motional narrowing model results in DBV estimates that are closest to what is expected from the literature.

Another supplementary comparison of the L, 1C, and 2C models was carried out on the same data with only 11 *τ* values. Inter-subject grey matter average parameter estimates are shown in Supplementary Fig. S3. There a statistically significant difference (*p* < 0:001) between OEF estimates made with the L model on 11-*τ* versus 24-*τ* data, but no significant difference between estimates made with the 1C or 2C models, suggesting that these more complete models are more robust to changes introduced by undersampling in *τ*.

Supplementary Table S1 (which parallels Table 3) shows the results of inference using the 2C model, with an assumed *Hct* of 0.34. DBV estimates were higher under the spatially-regularized 2C model with *Hct* = 0:34, which is to be expected given the dependence on *Hct* in the intravascular compartment model’s field inhomogeneity parameter *G*_0_ (Equation (11)). OEF estimates were also higher with a lower *Hct* for the 2C model, both with and without spatial regularization. As expected, there was no difference in $R_{2}^{\overset{\overset{’}{}}{}}$ or DBV estimates based on *Hct* using the L or 1C models (not shown). Therefore, the OEF estimates from the L or 1C model were scaled as the ratio of the *Hct* values i.e. ~17% increase in OEF with *Hct* = 0:34.

Median grey matter free energy was used to compare the goodness of fit with each regularization method. Group-average free energy was −235 ± 53 and −208 ± 51 for 1C model (without and with spatial regularization, respectively), and −230 ± 58 and −207 ± 50 for 2C. Both these differences were statistically significant with *p* ≥ 10^−3^. There were no significant differences in free energy between the two models when the same regularization was used.

## 5 Discussion

### 5.1 Improvements to model fitting using simulated data

Previously published work (Christen et al., 2014; Lee et al., 2018) has shown that the qBOLD model is not optimally suited for simultaneous estimation of OEF and DBV, which are important parameters in research or clinical assessment of brain metabolism. In this study, simulated data was used to demonstrate the collinearity between OEF and DBV that makes accurate estimation difficult. It also showed that the likelihood distribution in $R_{2}^{\overset{\overset{’}{}}{}}$-DBV space (Fig. 1b) has visibly lower correlation between the parameters, providing the opportunity to accurately estimate them simultaneously. Furthermore, the distribution is relatively smooth and symmetrical in both the $R_{2}^{\overset{\overset{’}{}}{}}$ and DBV dimensions, so the assumption of a multi-variate normal distribution is reasonable, making these parameters suitable for VB analysis.

Simulated ASE-qBOLD data were used to optimise parameters for VB inference. In particular, prior standard deviations were chosen that resulted in the most accurate estimation across a broad range of OEF values (from 20% to 70%, encompassing both healthy and pathological values (Marchal et al., 1992)) and DBV values (from 0.3% to 15% (Roland et al., 1987)). The prior mean values were chosen based on relevant prior work (Stone and Blockley, 2017). It would also be possible to use values from other imaging modalities to define prior means. For example, a hyperoxia experiment could be used to estimate venous cerebral blood volume in grey matter (Blockley et al., 2013), which could be used to inform the prior on DBV. It was, however, shown that significantly changing the prior means does not have a detrimental effect on the accuracy of parameter estimation. This is to be expected given the deliberate choice of broad standard deviations for the priors.

Simulated data with a range of SNRs were also used to compare least-squares fitting of a log-linear model with VB inference on the asymptotic qBOLD model with one or two compartments. The model used for the second (intravascular) compartment is one that accounts for the fact that spins inside blood vessels diffuse over a significantly greater distance than the size of a red blood cell in TE, so they are in the motional narrowing regime (Berman and Pike, 2017). Fig. 3 showed that VB inference using the 1C or 2C models produced OEF estimates that were significantly more accurate than the L model at SNRs below 100, and estimated both OEF and DBV more accurately than the 1C and 2C models fit using least squares regression. Though LS regression of the 2C model estimated $R_{2}^{\overset{\overset{’}{}}{}}$ more accurately than other methods, it also performed worse in DBV estimation, leading to poorer OEF estimates overall, compared with the VB implementation of the same model.

Across all parameters, there was not a substantial difference in performance between the 1C and 2C models. This is likely to be because in voxels with normal DBV, the intravascular compartment contributes a very small amount to the overall signal, and so could potentially be ignored. Similarly, additional analysis showed that the 1C and 2C models were more accurate at estimating $R_{2}^{\overset{\overset{’}{}}{}}$ and OEF on sparser data, particularly at low SNR (see Supplementary Fig. S1). The difference in OEF error between L and 2C models in Fig. S1 is greater than in Fig. 3., suggesting that the use of this analysis framework is even more important when used with shorter acquisition times, such as in a clinical protocol.

One of the limitations of this study is the use of the analytical qBOLD model as the ground truth. Generation of synthetic qBOLD signals using a more detailed model incorporating different vascular compartments and vessel sizes would be more representative of *in vivo* data. Future work will concentrate on the integration of such models to validate and optimise new sqBOLD implementations.

### 5.2 *In vivo* validation

The same analyses were applied to GASE data from 7 healthy subjects, and grey matter mean parameter estimates were compared at the group level. Statistically significant differences were found between the L model and 1C and 2C models in $R_{2}^{\overset{\overset{’}{}}{}}$ and OEF estimation (but not between 1C and 2C), and between the L model and 1C model (but not 2C) in DBV estimation. The grey matter inter-voxel standard deviations of all parameters were significantly lower in VB estimation than the L model. This suggests that VB inference with a more complete model is less susceptible to fitting extreme values. Inter-voxel standard deviations are higher than the errors reported for simulated data of the same SNR because they also account for physiological variation across grey matter. The number of voxels with un-physiological values was reduced when using the 2C model compared with the 1C model, suggesting that modelling the blood signal provides an improvement to overall model fitting. The choice of intravascular signal model within the 2C model affected estimates of DBV, but not of $R_{2}^{\overset{\overset{’}{}}{}}$ or OEF, at the group level. The motional narrowing model was preferred, because it has been shown from a theoretical perspective to accurately model signal evolution in the blood following a refocussing pulse (Berman and Pike, 2017).

The expectation of spatial homogeneity of parameters can be incorporated into VB analysis using spatial regularization, in which the estimated posterior distributions of adjoining voxels are used as priors in an iterative process. The effect of spatial regularization was tested in both the 1C and 2C models. In both cases, though there was not a significant change in group-level parameter averages, the median free energy increased significantly, suggesting a better fit of the model to the signal. The resulting parameter maps (exemplified in Fig. 4) were also more homogeneous, and had fewer voxels where parameters had been estimated as having un-physiological values (such as OEFs above 100%).

The parameter estimates obtained from all methods are susceptible to partial volume effects from infiltrating white matter (where the assumptions of the qBOLD model do not hold), especially given the large voxel size used. By defining the grey matter segmentation threshold more strictly, it is possible to reduce this effect and to obtain a more accurate grey matter average, although the whole-brain maps still contain mixed voxels where estimates are not accurate. A model that describes the qBOLD signal in white matter could be used alongside a partial volume correction paradigm, as used in the analysis of arterial spin labelling data (Chappell et al., 2011), to correct for this effect.

### 5.3 Comparison of parameter estimates with literature values

Parameter estimates compared with literature values can be found in Table 4. The average grey matter $R_{2}^{\overset{\overset{’}{}}{}}$ estimate of 3.7 ± 0.4s^−1^ was higher than the 2.9 ± 0.4s^−1^ reported by He and Yablonskiy (2007) and the 3.1 ± 0.3s^−1^ reported by Simon et al. (2016). However, both of those apply the qBOLD model to GESSE data, which requires simultaneous quantification of *R*_2_ and $R_{2}^{\overset{\overset{’}{}}{}}$, whereas the GASE data used here is not sensitive to *R*_2_.

Grey matter DBV was here estimated to be 7.00 ± 0.55%, which is significantly higher than reported elsewhere, although there is considerable variation in the literature, with DBV ranging from 1.75 ± 0.13% (He and Yablonskiy, 2007) to 4.9 ± 2.0% (Lee et al., 2018) or up to 5% (An and Lin, 2003). This may be due to uncertainty introduced by assuming values of parameters used to calculate actual DBV from apparent DBV (Equation (12)), or due to the signal transient often observed at *τ* = 0 (He and Yablonskiy, 2007, see, in particular, Fig. 1C). Here, signal is consistently higher at *τ* = 0 than would be expected, which may lead to underestimation of apparent DBV in the linear model. The 1C and 2C models, which are not dependent on a single data point for their DBV estimation, should be more robust against this error than the L model. The 2C model, had less inter-subject variation in DBV estimates than the 1C model.

OEF estimates obtained using sqBOLD (with any analysis method) are significantly lower than those obtained using GESSE-qBOLD (He and Yablonskiy, 2007; Domsch et al., 2018) or non-qBOLD methods such as TRUST (Lu and Ge, 2008) and oxygen-15 tracer based PET (Derdeyn et al., 2001). This is in part due to the over-estimation of DBV that is consistently reported in ASE-based qBOLD studies. It may also be due to the assumed value of *Hct* = 0.40. The supplementary analysis with *Hct* = 0.34 led to an increase in estimated OEF to 21% (with the 2C model), which is closer to literature values, but was accompanied by an increase in estimated DBV to 8.4%, which is even further from what would be expected. The exact value of average *Hct* in GM is not known, although a reduction of around 15% from arterial *Hct* (around 0.40) is supported by Calamante et al. (2016). It is possible that larger veins (with *Hct* = 0.40) may contribute more to the ASE qBOLD signal than smaller vessels with lower *Hct*. Similarly, this study assumed Δ*χ*_0_ = 0:264 ppm (Spees et al., 2001) as opposed to Δ*χ*_0_ = 0:18 ppm used in older studies (An and Lin, 2003). However, despite this systematic offset it has been demonstrated elsewhere that OEF estimates using this experimental technique are modulated by pathology (Stone et al., 2019).

OEF overestimation may also be due to error in $R_{2}^{\overset{\overset{’}{}}{}}$ estimation, which could be caused by diffusion in the tissue compartment, which is ignored in the static dephasing model (Yablonskiy and Haacke, 1994). Simulation and *in vivo* studies have shown that diffusion of water in brain parenchyma affects the BOLD signal measured under the GESSE sequence (Kiselev and Posse, 1999; Dickson et al., 2010) and ASE (Ni et al., 2015). A qBOLD model which accounts for diffusion in ASE, like that constructed by Dickson et al. (2010) may enable more accurate estimation of $R_{2}^{\overset{\overset{’}{}}{}}$ and DBV, although it requires quantification (or assumption) of the rate of diffusion, and the distribution of vessel sizes in grey matter. The GESEPI acquisition used (Yang et al., 1998; Blockley and Stone, 2016) reduces the effect of magnetic field inhomogeneities, which improves the physiological specificity of $R_{2}^{\overset{\overset{’}{}}{}}$ estimation, but does not perfectly remove them, which may explain why DBV estimates are higher than those obtained after retrospective magnetic field gradient correction (Yablonskiy, 1998).

The clinical utility of OEF estimation is primarily in quantitative inter-subject and inter-regional comparisons. The group level standard deviation of OEF among young healthy subjects reported here was similar to, or smaller than, those of other methods. Normalized variance, accounting for differences in mean OEF, is lower under spatially regularized VB than other methods. This suggests that the 2C, spatially regularized method proposed here may be useful in identifying differences across a population. Similarly, OEF is expected to be uniform across the healthy brain, and the spatially regularized 1C and 2C models produced smooth maps. These methods may, therefore, be useful in identifying regions of altered OEF, such as the ischaemic penumbra, by comparison with healthy tissue.

Other literature methods required precise initialization of parameter values in order to obtain reasonable results after model fitting. This is not required in a Bayesian framework, as broad, minimally-informative priors can be used to produce accurate results across a range of parameter values, as was shown in simulated data. Model-based fitting of sqBOLD data does, therefore, have considerable potential utility, although there are significant limitations which must be investigated further.

## 6 Conclusions

This study has shown that a Bayesian framework can be used to estimate parameters in the qBOLD model reliably and consistently, when compared with previous analysis methods. It provides additional information in the form of parameter uncertainties, allows for the possibility of using priors to inform estimates, and can incorporate spatial regularization which improves parameter distributions. The non-linear model used can be extended in a number of ways, such as including contributions to the signal from the intravascular compartment. The resulting parameter maps of $R_{2}^{\overset{\overset{’}{}}{}}$ and DBV can be used to quantify OEF, whose spatial distribution signifies clinically useful information, especially in the case of acute stroke, where it could be used to distinguish the ischemic penumbra from the ischemic core (Hossmann, 1994; Liu and Li, 2016). Grey matter average parameter estimates obtained using VB have significantly lower variation than those of the simpler model, leading to more homogeneous distributions and more precise average values. The distributions are even more homogeneous, and the average values more precise, when using a model that includes the intravascular signal contribution.

It has also been shown that ASE-qBOLD consistently estimates parameter values that are different from those obtained through other methods. In particular, DBV estimates tend to be higher than literature values, leading to lower OEF estimates. These differences could be the result of motional narrowing effects in the tissue compartment. Further work is required to determine whether these measurements can provide clinically relevant information without further correction. In addition, alternative qBOLD techniques, including those that use data acquired by other modalities such as GESSE, could be analysed in the same VB framework, in order to produce a comparison between the various methods for measuring $R_{2}^{\overset{\overset{’}{}}{}}$ (and hence, OEF) *in vivo*. Comparing other methods of OEF estimation (such as TRUST), or DBV estimation (using hyperoxia BOLD), in the same subjects, would also be very useful for validation.

The method presented here estimates parameters more accurately from simulated data, and leads to significantly less variance at the intrasubject level *in vivo*. Its utility could be reinforced by being applied to GASE data from clinical populations such as acute stroke patients, in order to distinguish between regions of the brain with varying OEF.

## Supplementary Material

Supplementary material to this article can be found online at https://doi.org/10.1016/j.neuroimage.2019.116106.

Suppl figs1-3, suppl table 1

## Figures and Tables

**Table 1:** Parameter values used for simulating data.

Parameter	Value	Reference
Hct	0.40	Nicoll et al. (2012)
Δχ_0_	0.264 ppm	Spees et al., 2001
$R_{2}^{t}$	11.5 s^−1^	He & Yablonskiy (2007)
$R_{2}^{b,\text{true}}$	5.29 s^−1^	Berman et al. (2018)
t_D_	4.51ms	Berman et al. (2018)
B_0_	3 T	
TE	74 ms	
TR	3 s	
*τ* range	−28 to 64 ms	
*τ* step size	4ms	

**Table 2:** FABBER Variational Bayes parameters, with their prior means and standard deviations, and the means and standard deviations used to initialize the approximate posterior. Mean values for $R_{2}^{’}$ and DBV are taken from Stone and Blockley (2017). Other model parameters are fixed to assumed values (Table 1).

Parameter	Prior		Initial Posterior	
	Mean	Standard Deviation	Mean	Standard Deviation
DBV (%)	3.6	10^3/2^	3.6	10^3/2^
$R_{2}^{’}$ (s^−1^)	2.6	10^3/2^	2.6	10^3^
	500	10^3^	500	10^3^

**Table 3:** Parameter estimates of $R_{2}^{’},$ DBV, and OEF using three qBOLD models: linear model (L) considering only tissue signal at long-*τ* and *τ* = 0 data-points; a one-compartment model (1C) considering tissue signal across all *τ*; and a two-compartment model (2C) that includes intravascular signal. Both 1C and 2C models include spatial regularization. The table shows grey matter mean ± grey matter inter-voxel standard deviation, for 7 healthy subjects, and group mean ± group standard deviation.

Model	${\text{R}}_{2}^{’}\left({\text{s}}^{-1}\right)$					DBV (%)					OEF (%)				
	L	1C	1C	2C	2C	L	1C	1C	2C	2C	L	1C	1C	2C	2C
Regularization	None	None	Spatial	None	Spatial	None	None	Spatial	None	Spatial	None	None	Spatial	None	Spatial
1	3.1±1.9	3.6±1.1	3.6±1.0	3.6±1.3	3.6±1.0	4.9±3.9	6.7±3.2	6.7±3.6	6.4±3.0	6.4±3.5	24±17	19±11	22±12	20±11	23±13
2	3.7±2.8	3.9±1.4	4.2±1.4	4.0±1.8	4.2±1.4	6.3±5.4	7.4±4.0	7.6±4.4	7.1±3.8	7.1±4.2	20±14	17±11	18±11	17±11	20±12
3	3.0±2.7	3.2±1.1	3.4±1.2	3.5±1.7	3.5±1.2	4.4±3.9	6.6±3.1	6.6±3.4	6.4±2.9	6.4±3.2	23±17	16±11	19±12	17±11	19±12
4	3.5±3.2	3.9±1.3	4.3±1.3	4.1±1.8	4.4±1.4	5.4±5.1	7.3±3.4	7.5±3.8	6.7±3.1	7.4±3.7	21±14	17±10	17±10	19±11	18±11
5	3.1±2.9	3.4±1.4	3.4±1.5	3.9±2.1	3.3±1.8	5.8±5.4	6.9±3.4	6.9±3.6	6.1±3.0	6.8±3.6	20±14	16±11	18±12	18±12	20±14
6	3.5±2.9	3.9±1.1	4.1±1.2	4.0±1.4	4.2±1.2	5.8±5.8	6.6±3.3	6.5±3.6	6.0±2.9	6.5±3.6	23±17	19±12	20±12	21±12	22±12
7	2.8±2.6	2.9±1.4	3.0±1.8	3.5±2.7	2.8±2.0	5.2±5.1	6.1±3.5	6.2±3.7	5.8±3.1	6.6±3.7	18±15	16±13	16±13	16±15	18±15
**Mean**	**3.2±0.3**	**3.5±0.4**	**3.8±0.4**	**3.8±0.4**	**3.6±0.4**	**5.4±0.6**	**6.8±0.4**	**6.9±0.5**	**6.4±0.4**	**6.7±0.4**	**21±2**	**17±2**	**19±2**	**18±2**	**20±2**

**Table 4:** Group grey-matter average parameter values for $R_{2}^{’}$, DBV, and OEF, showing group mean ± group standard deviation, with comparisons with other methods from the literature.

Method	${\text{R}}_{2}^{’}$ (s^−1^)	DBV (%)	OEF (%)
Log-Linear qBOLD	3.2±0.3	5.39±0.62	21.4±2.1
1C qBOLD (spatially regularized)	3.8±0.3	7.59±0.70	19.2±1.6
2C qBOLD (spatially regularized)	3.7±0.4	7.00±0.55	19.1±1.8
qBOLD (He and Yablonskiy, 2007)	2.9±0.4	1.75±0.13	38.3±5.3
qBOLD (Simon et al., 2016)	3.1±0.4	–	–
GESFIDE (Ni et al., 2015)	2.7±0.4	–	–
GESSE (Ni et al., 2015)	2.7±0.3	–	–
Neural network qBOLD (Domsch et al., 2018)	–	4.2±0.1	40±1
Conventional qBOLD (Lee et al., 2018)	–	4.9±2.0	31.8±6.0
Interleaved qBOLD (Lee et al., 2018)	–	3.1±0.5	39.9±3.3
TRUST (Lu and Ge, 2008)	–	–	35±6
PET (Derdeyn et al., 2001)	–	–	41±9	41±9
