# Supplementary material for: Model-based Bayesian inference of brain oxygenation using quantitative BOLD
Source: Neuroimage. Author manuscript; Available in PMC 2020 Jul 3. (PMC7334042; doi:10.1016/j.neuroimage.2019.116106)
Supplement: Suppl figs1-3, suppl table 1 [file EMS86708-supplement-Suppl_figs1_3__suppl_table_1.docx]

# Appendix A. Data Access Statement

The raw data that underpins this work can be accessed via the Oxford Research Archive doi: <http://dx.doi.org/10.5287/bodleian:6R5px9K0X>. MATLAB code used for simulations, and for analysis and presentation of inference results, can be accessed via Zenodo, doi: <https://doi.org/10.5281/zenodo.3243308>.

# Appendix B. Supplementary Material

**Figure S1.** Error in parameter estimates of **a)** $R_{2}^{'}$, **b)** DBV, and **c)** OEF as a function of SNR, for each model (L, 1C, and 2C) using simulated data with 11 $\tau$ values from -16 ms to 64 ms (in steps of 8 ms), as used in Stone et al., 2019. Results are comparable in $R_{2}^{'}$ and DBV estimation between all three models, but the 1C and 2C models perform better at OEF estimation, especially at low SNR.

**Figure S2.** Group average grey matter estimates of **a)** $R_{2}^{'}$, **b)** DBV, and **c)** OEF, with error bars indicating inter-subject standard deviation, for the 2C model using Linear (Equation 7), Powder (Equation 8) and motional narrowing (M.N.R - Equation 10) models for the intravascular signal. Two-way ANOVA and pair-wise comparisons show that there is no significant difference in estimates of $R_{2}^{'}$ or OEF, but that all three methods result in different average DBV estimates.

**Figure S3.** Group average grey matter estimates of **a)** $R_{2}^{'}$, **b)** DBV, and **c)** OEF, with error bars indicating inter-subject standard deviation, for L, 1C, and 2C models (with VB inference, without spatial regularization) on data with 11 $\tau$ values (from -16 ms to +64 ms, in steps of 8 ms). The L model is significantly different from both 1C and 2C in all parameters. Estimates of $R_{2}^{'}$ and OEF from 1C and 2C models are not significantly different, but estimates of DBV are. The 1C model estimates DBV to be much higher than the other models.

|  | $\boldsymbol{R}_{\boldsymbol{2}}^{\boldsymbol{'}}$ **(s^-1^)** | |  | **DBV (%)** | |  | **OEF (%)** | |
| --- | --- | --- | --- | --- | --- | --- | --- | --- |
| **Model** | **2C** | **2C** |  | **2C** | **2C** |  | **2C** | **2C** |
| **Regularization** | **None** | **Spatial** |  | **None** | **Spatial** |  | **None** | **Spatial** |
| **1** | 3.7$\pm$2.4 | 3.9$\pm$2.3 |  | 6.6$\pm$3.3 | 8.1$\pm$2.6 |  | 22$\pm$15 | 22$\pm$14 |
| **2** | 4.0$\pm$3.5 | 4.3$\pm$3.3 |  | 6.8$\pm$5.1 | 9.4$\pm$3.5 |  | 20$\pm$17 | 20$\pm$15 |
| **3** | 3.4$\pm$3.2 | 3.4$\pm$3.2 |  | 5.9$\pm$3.6 | 7.5$\pm$3.0 |  | 20$\pm$16 | 21$\pm$15 |
| **4** | 4.1$\pm$3.6 | 4.4$\pm$3.8 |  | 6.4$\pm$4.4 | 9.3$\pm$3.8 |  | 22$\pm$17 | 20$\pm$15 |
| **5** | 3.5$\pm$3.2 | 3.7$\pm$3.5 |  | 6.0$\pm$5.6 | 8.7$\pm$4.0 |  | 20$\pm$18 | 19$\pm$16 |
| **6** | 4.1$\pm$3.7 | 4.2$\pm$3.6 |  | 7.1$\pm$4.9 | 8.3$\pm$4.2 |  | 19$\pm$17 | 23$\pm$16 |
| **7** | 3.5$\pm$3.0 | 3.0$\pm$3.3 |  | 5.5$\pm$3.7 | 7.1$\pm$3.0 |  | 20$\pm$16 | 19$\pm$15 |
| **Mean** | **3.6**$\pm$**0.4** | **3.9**$\pm$**0.5** |  | **6.3**$\pm$**0.5** | **8.4**$\pm$**0.9** |  | **21**$\pm$**1** | **21**$\pm$**1** |

**Table S1.** Parameter estimates of $R_{2}^{'}$, DBV, and OEF using the 2C qBOLD model, with $Hct$ fixed to 0.34 (as opposed to $Hct$=0.40 as in Table 4). The table shows grey matter mean $\pm$ grey matter inter-voxel standard deviation, for 7 healthy subjects, and group mean $\pm$ group standard deviation. The results of $R_{2}^{'}$ and DBV estimates for the L and 1C models (not shown) are the same as those in Table 4.
